# A rare presentation of high-grade serous ovarian carcinoma with ulcerating inguinal lymph node metastasis: a case report

**DOI:** 10.1093/jscr/rjaf317

**Published:** 2025-05-21

**Authors:** Hassan M Latifah, Ruzanah Almarzugi, Hussam Bitar, Mohammad Alyafi, Abdulmalik Abumohssin, Zuhoor Almansouri, Batool Abdulaziz Kabli, Nusaybah A Shafi, Saeed Baradwan

**Affiliations:** Department of Obstetrics and Gynecology, King Faisal Specialist Hospital and Research Center, Jeddah 23433, Saudi Arabia; Department of Obstetrics and Gynecology, King Faisal Specialist Hospital and Research Center, Jeddah 23433, Saudi Arabia; Department of Surgery, King Faisal Specialist Hospital and Research Center, Jeddah 23433, Saudi Arabia; Department of Obstetrics and Gynecology, Faculty of Medicine, King Abdulaziz University, Jeddah 22254, Saudi Arabia; Department of Radiology, King Faisal Specialist Hospital and Research Center, Jeddah 23433, Saudi Arabia; Department of Pathology and Laboratory Medicine, King Faisal Specialist Hospital and Research Center, Jeddah 23433, Saudi Arabia; College of Medicine, Umm Al-Qura University, Makkah 21955, Saudi Arabia; College of Medicine, Umm Al-Qura University, Makkah 21955, Saudi Arabia; Department of Obstetrics and Gynecology, King Faisal Specialist Hospital and Research Center, Jeddah 23433, Saudi Arabia

**Keywords:** high grade epithelial ovarian cancer, inguinal lymph node, lymphatic spread, distant metastasis

## Abstract

High-grade serous ovarian carcinoma (HGSOC) typically spreads within the peritoneal cavity, and metastatic spread to extraperitoneal lymph nodes, particularly the inguinal region, is rare. Herein, we present a case of a 47-year-old premenopausal woman who presented with an enlarging, ulcerating right inguinal mass. Imaging revealed a necrotic inguinal mass, with no significant intra-abdominal findings. Surgical excision was performed, and histopathology confirmed metastatic HGSOC. She then underwent neoadjuvant chemotherapy, followed by radiotherapy. She then underwent robotic-surgical staging. Histopathological analysis showed bilateral ovarian involvement with multiple tumor foci. Genetic testing identified a BRCA1 mutation, leading to adjuvant maintenance therapy with Olaparib. This case emphasizes the importance of considering ovarian carcinoma in the differential diagnosis of unusual inguinal lymph node masses, even without a visible ovarian mass. Early detection, genetic testing, and a multidisciplinary approach are essential for optimizing patient outcomes in advanced HGSOC with atypical metastasis.

## Introduction

High-grade serous ovarian carcinoma (HGSOC) is the most frequent and aggressive histologic subtype of epithelial ovarian cancer, repeatedly presenting at an advanced stage due to its insidious onset and lack of early symptoms [1]. The disease primarily spreads through transcoelomic dissemination within the peritoneal cavity, with frequent metastatic locations involving the omentum, peritoneum, and distant organs such as the liver and lungs [2]. However, extraperitoneal lymphatic spread, particularly to the inguinal lymph nodes, is an uncommon occurrence and is rarely registered in the literature [3].

Lymphatic dissemination of HGSOC typically follows a retroperitoneal pathway, involving the pelvic and para-aortic lymph nodes [2]. Inguinal lymph node metastasis, especially presenting as an ulcerated mass, is exceedingly rare [3] and poses a diagnostic challenge, often mimicking other malignancies or primary cutaneous lesions. This atypical presentation may lead to delays in diagnosis and modifications in management strategies.

In this case report, we present an unusual manifestation of HGSOC, characterized by an ulcerating inguinal lymph node metastasis in a patient with advanced disease but no gross ovarian mass on imaging. Through this report, we aim to highlight the diagnostic challenges, possible pathophysiologic mechanisms, and clinical implications of this rare metastatic pattern.

## Case report

A 47-year-old premenopausal nulliparous woman with an unremarkable medical history presented with a progressively enlarging, painful right inguinal mass. She denied constitutional symptoms such as weight loss, night sweats, or fever. On examination, the mass measured 8 × 5 × 7 cm, was fungating, fixed to surrounding structures, and exhibited minimal bleeding.

**Figure 1:**
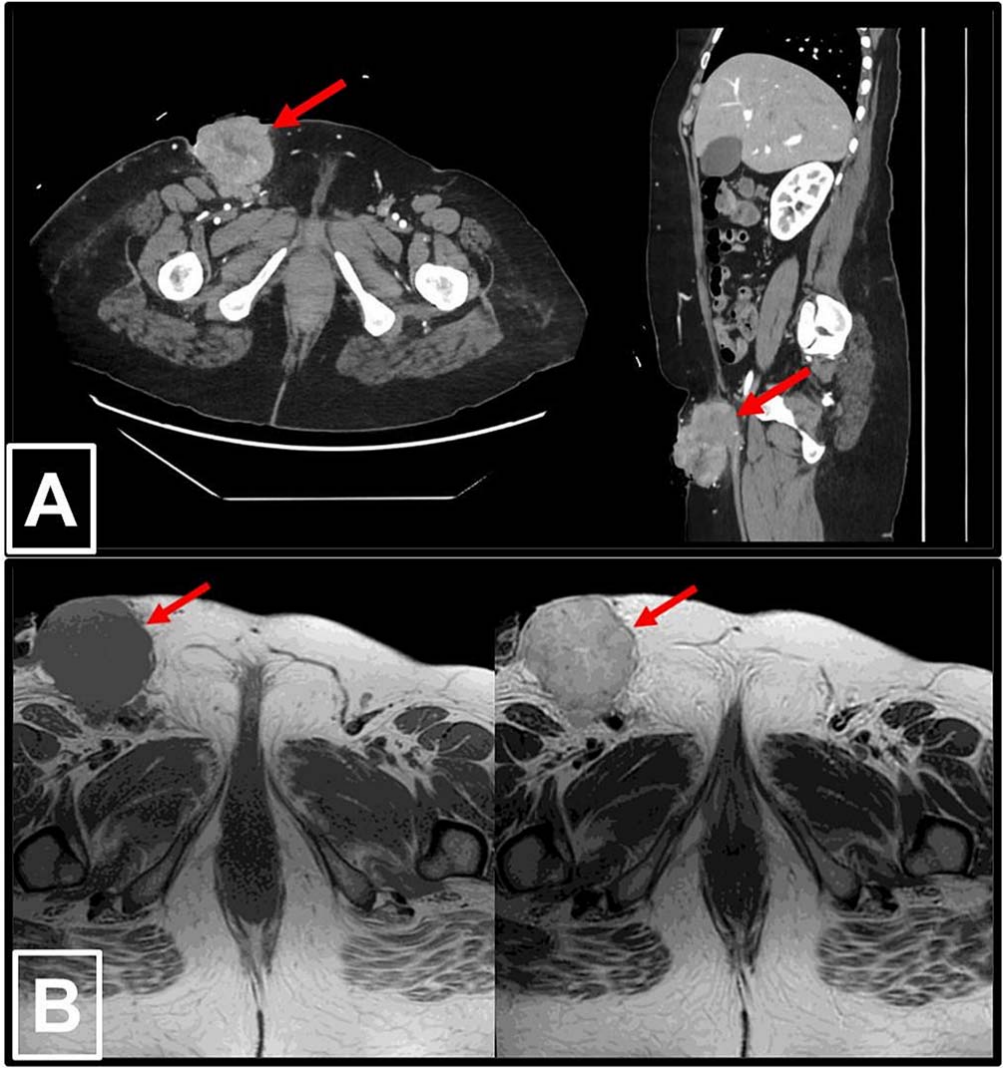
Radiological imaging of the right inguinal mass. (a) Contrast-enhanced CT scans of the abdomen and pelvis in axial (left) and sagittal (right) views, demonstrating a pathologically enlarged necrotic right inguinal lymph node (red arrow), measuring 5.3 × 6.2 × 8.5 cm. The mass is ulcerating into the inguinal skin and is in very close proximity to the adjacent inguinal vessels. (b) MRI of the pelvis in T1-weighted (left) and T2-weighted (right) sequences, again revealing the pathologically enlarged necrotic right inguinal lymph node (red arrow).

Laboratory tests showed an elevated CA-125 level of 531.7 U/mL. Contrast-enhanced computed tomography (CT) and magnetic resonance imaging (MRI) of the abdomen and pelvis depicted a large necrotic right inguinal mass measuring 5.3 × 6.2 × 8.5 cm, ulcerating into the skin, and closely abutting adjacent inguinal vessels. No significant intra-abdominal lymphadenopathy or thoracic metastases were detected (Fig. 1).

**Figure 2:**
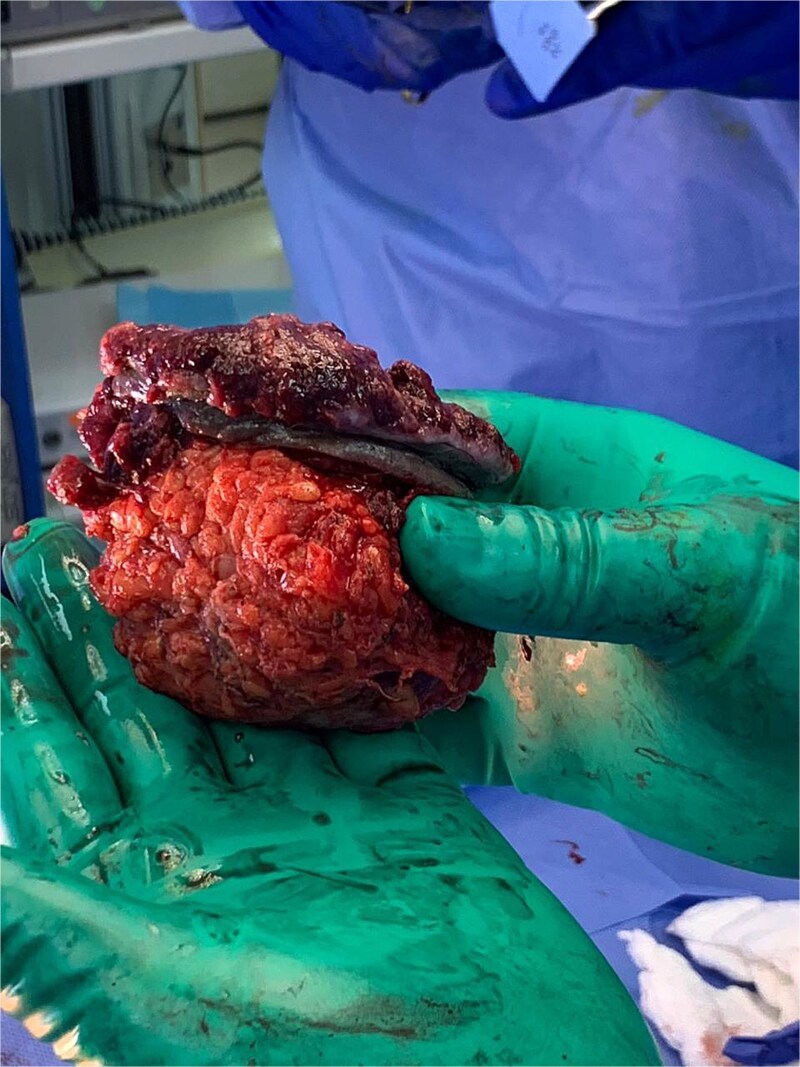
Gross image of the partially resected right inguinal mass showing nodularity and ulceration.

**Figure 3:**
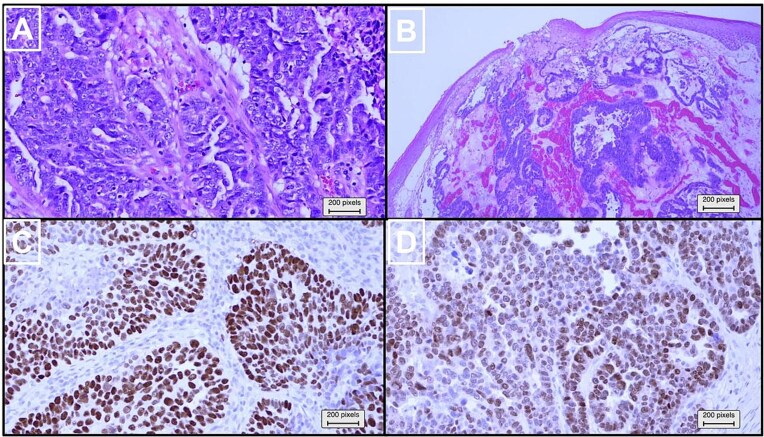
Histopathological and immunohistochemical analysis of the partially resected inguinal mass. (a) Higher magnification of hematoxylin and eosin (×100) revealed pleomorphic neoplastic cells with a high nuclear-to-cytoplasmic ratio, prominent nucleoli, and frequent mitoses. (b) Hematoxylin and eosin (×20) stained slides showed infiltrative carcinoma with glandular, cribriform, and solid growth patterns, overlaid by skin. Immunohistochemical analysis (×100) revealed that the tumor cells stained positive for (c) TP53 and (d) PAX8.

Given the nature of the mass, the patient underwent urgent surgical excision (Fig. 2). However, Due to the tumor abutting the femoral vessels, complete resection was not possible. Histopathological examination established metastatic high-grade serous carcinoma of Müllerian origin, with tumor cells positive for CK7, PAX8, P53 (diffuse and strong), and P16 (diffuse and strong), while negative for CK20, CDX2, TTF1, and GATA3 (Fig. 3). An endometrial biopsy demonstrated weakly proliferative endometrium with reactive changes, and a pap smear was negative for malignancy.

Following confirmation of metastatic ovarian carcinoma, the patient received six cycles of neoadjuvant combinational chemotherapy of paclitaxel and carboplatin, followed by radiotherapy. After 6 months of treatment, her CA-125 level decreased to 7.8 U/mL. She subsequently underwent robotic-assisted total abdominal hysterectomy, bilateral salpingo-oophorectomy, right pelvic lymph node dissection, and infracolic omentectomy with peritoneal washing. The intraoperative findings clearly indicated markedly enlarged right pelvic nodes, with the largest measuring 3.5 × 2.3 cm. These nodes were successfully resected during the surgical procedure.

Histopathological analysis revealed bilateral ovarian involvement with multiple tumor foci, the largest measuring 0.8 cm, but no surface invasion. The right fallopian tube also showed tumor involvement. The lymph nodes examined were negative for malignancy and complete pathological response following treatment with chemotherapy and radiotherapy. However, atypical cells were detected in the pelvic washings.

Genetic testing confirmed a BRCA1 mutation, prompting the tumor board to recommend adjuvant maintenance therapy with olaparib under the supervision of the medical oncology team. Additionally, the patient was referred to breast surgery for evaluation of a risk-reducing bilateral mastectomy. Ongoing surveillance by the gynecologic oncology team included regular CA-125 monitoring and imaging as needed. At 6 months, there was no clinical or radiological proof of relapse, and the patient was doing well.

## Discussion

HGSOC is an aggressive malignancy that frequently presents at an advanced stage, often with peritoneal carcinomatosis and widespread intraperitoneal dissemination. While retroperitoneal lymph node involvement, particularly of the pelvic and para-aortic nodes, is a well-recognized feature of HGSOC [2], extraperitoneal lymphatic spread is uncommon [3]. This case report highlights a rare presentation of HGSOC with an ulcerating inguinal lymph node metastasis, an atypical pattern of dissemination that poses diagnostic and therapeutic challenges.

The presence of an ulcerating inguinal lymph node mass in a patient without a known primary malignancy often leads to a broad differential diagnosis, including cutaneous malignancies such as squamous cell carcinoma or melanoma, primary lymphomas, and metastatic disease from gynecologic, gastrointestinal, or genitourinary cancers [4]. In this case, the absence of an obvious ovarian mass on imaging further complicated the diagnostic process, necessitating histopathological and immunohistochemical confirmation to establish the Müllerian origin of the tumor.

Inguinal lymph node metastasis from ovarian carcinoma is extremely rare due to the typical routes of HGSOC spread, which include transcoelomic dissemination within the peritoneal cavity and retroperitoneal lymphatic spread [2]. Diagnosing metastatic ovarian carcinoma in an inguinal lymph node is particularly difficult when no ovarian mass or peritoneal disease is present. In these cases, immunohistochemical markers are essential for a definitive diagnosis. Positive expression of CK7, PAX8, and p53, along with the absence of CK20, CDX2, and TTF1, provides strong evidence for high-grade serous carcinoma of Müllerian origin [5].

Lymphatic spread in HGSOC typically follows a stepwise pattern, involving the pelvic, para-aortic, and supradiaphragmatic lymph nodes [2]; however, alternative lymphatic pathways may occasionally lead to the rare inguinal metastasis [3]. Possible mechanisms for this rare dissemination include (i) aberrant lymphatic drainage, where tumor cells bypass the usual retroperitoneal routes due to anomalous connections between the peritoneal and inguinal lymphatic systems; (ii) lymphovascular invasion with retrograde spread, particularly when primary lymphatic pathways are obstructed; and (iii) direct tumor extension along lymphovascular structures or soft tissues, facilitating inguinal involvement. While these mechanisms remain speculative, the presence of an ulcerated inguinal mass in the current case suggests an advanced disease process with extensive lymphovascular dissemination. The tumor’s involvement of the inguinal vessels further supports the possibility of direct lymphovascular invasion as a contributing factor.

The involvement of inguinal lymph nodes in HGSOC is classified as distant metastasis [International Federation of Gynecology and Obstetrics (FIGO) Stage IVB], significantly altering the prognosis and therapeutic considerations [6]. Patients with extraperitoneal metastases typically have a poorer prognosis due to the more extensive disease burden and increased likelihood of treatment resistance [7]. However, despite the advanced stage, this patient demonstrated an excellent response to chemotherapy, with normalization of CA-125 levels and no residual tumor after surgical intervention. Several investigations [8, 9] have shown that ovarian cancer patients classified as stage IVB exclusively due to inguinal lymph node metastases have survival rates comparable to those with para-aortic or pelvic lymph node involvement and significantly better outcomes than patients with distant organ metastases. Therefore, the authors of this case report do not support reclassifying these patients as stage IVB.

The presence of a BRCA1 mutation in this patient is of particular significance, as BRCA-mutated HGSOC is often more responsive to platinum-based chemotherapy and poly (ADP-ribose) polymerase (PARP) blockers [10]. This underscores the vital role of genetic testing in treatment planning and identifying candidates for targeted therapies like olaparib, which has been shown to significantly improve progression-free survival (PFS) in these patients [11].

Managing HGSOC with atypical metastases requires a multimodal approach, incorporating surgery, chemotherapy, and targeted therapy. In this case, the ulcerating and vascularly abutted inguinal mass precluded complete resection at initial presentation, necessitating neoadjuvant chemotherapy to reduce tumor burden before definitive cytoreductive surgery. The patient was managed with a standard regime of paclitaxel plus carboplatin, which remains the first-line therapy for advanced HGSOC [12], demonstrating a significant reduction in CA-125 levels and radiologic tumor response. While radiotherapy is not yet recognized as a standard treatment for HGSOC [13], it deserves serious consideration for its potential to target residual tumors that are in close proximity to femoral vessels and are deemed inoperable. Given the presence of a BRCA1 mutation, maintenance therapy with the PARP inhibitor olaparib was initiated, as it has been illustrated to increase PFS in BRCA-mutated HGSOC [11]. Additionally, the BRCA1 mutation prompted discussions regarding risk-reducing strategies, including prophylactic bilateral mastectomy, highlighting the importance of genetic counseling and long-term surveillance in comprehensive patient management [14].

Given the advanced stage at diagnosis, rigorous long-term follow-up is vital for promptly identifying recurrence and managing the disease. Regular monitoring of CA-125 levels is crucial, as it helps track progression despite its limitations as a definitive marker. Periodic imaging, such as CT scans and MRIs, is essential for detecting recurrence, especially in atypical metastatic patterns. Additionally, thorough clinical assessments during follow-up visits are critical for uncovering early signs of recurrence. This proactive approach ensures timely intervention and significantly enhances patient outcomes.

## Conclusion

This case highlights the prominence of contemplating ovarian carcinoma in the differential diagnosis of ulcerating inguinal lymph node masses, even in the absence of a detectable ovarian mass. Furthermore, the role of BRCA1 mutation in guiding therapy underlines the necessity for genetic checking in all patients with HGOSC. This case also highlights the evolving landscape of ovarian cancer treatment, where the integration of targeted therapies and individualized treatment strategies is improving survival and quality of life for patients with advanced disease.

## References

[1] Punzon-Jimenez P, Lago V, Domingo S, et al. Molecular Management of high-grade serous ovarian carcinoma. Int J Mol Sci 2022;23:13777. 10.3390/ijms232213777 PMC9692799 36430255

[2] Bayraktar E, Chen S, Corvigno S, et al. Ovarian cancer metastasis: looking beyond the surface. Cancer Cell 2024;42:1631–6. 10.1016/j.ccell.2024.08.016 39270645

[3] Stavros S, Potiris A, Machairiotis N, et al. Inguinal lymph node metastasis as sole manifestation of ovarian/fallopian tube cancer: a review of the literature. J Cancer 2023;14:3176–81. 10.7150/jca.88863 37928416 PMC10622991

[4] Zaren HA, Copeland EM 3rd. Inguinal node metastases. Cancer 1978;41:919–23. 10.1002/1097-0142(197803)41:3<919::AID-CNCR2820410320>3.0.CO;2-A 638977

[5] Santoro A, Angelico G, Travaglino A, et al. The multiple facets of ovarian high grade serous carcinoma: a review on morphological, immunohistochemical and molecular features. Crit Rev Oncol Hematol 2025;208:104603. 10.1016/j.critrevonc.2024.104603 39732305

[6] Berek JS, Kehoe ST, Kumar L, et al. Cancer of the ovary, fallopian tube, and peritoneum. Int J Gynaecol Obstet 2018;143:59–78. 10.1002/ijgo.12614 30306591

[7] Deng K, Yang C, Tan Q, et al. Sites of distant metastases and overall survival in ovarian cancer: a study of 1481 patients. Gynecol Oncol 2018;150:460–5. 10.1016/j.ygyno.2018.06.022 30001833

[8] Nasioudis D, Chapman-Davis E, Frey MK, et al. Should epithelial ovarian carcinoma metastatic to the inguinal lymph nodes be assigned stage IVB? Gynecol Oncol 2017;147:81–4. 10.1016/j.ygyno.2017.07.124 28716307

[9] Hjerpe E, Staf C, Dahm-Kahler P, et al. Lymph node metastases as only qualifier for stage IV serous ovarian cancer confers longer survival than other sites of distant disease - a Swedish Gynecologic Cancer Group (SweGCG) study. Acta Oncologica (Stockholm, Sweden) 2018;57:331–7. 10.1080/0284186X.2017.1400691 29130381

[10] Paik J . Olaparib: a review as first-line maintenance therapy in advanced ovarian cancer. Target Oncol 2021;16:847–56. 10.1007/s11523-021-00842-1 34623572

[11] Tattersall A, Ryan N, Wiggans AJ, et al. Poly(ADP-ribose) polymerase (PARP) inhibitors for the treatment of ovarian cancer. Cochrane Database Syst Rev 2022;2:CD007929. 10.1002/14651858.CD007929.pub4 35170751 PMC8848772

[12] Ngoi NY, Syn NL, Goh RM, et al. Weekly versus tri-weekly paclitaxel with carboplatin for first-line treatment in women with epithelial ovarian cancer. Cochrane Database Syst Rev 2022;2:CD012007. 10.1002/14651858.CD012007.pub2 35188221 PMC8859866

[13] Macchia G, Titone F, Restaino S, et al. Is it time to reassess the role of radiotherapy treatment in ovarian cancer? Healthcare (Basel) 2023;11:2413. 10.3390/healthcare11172413 PMC10486999 37685447

[14] Bertozzi S, Londero AP, Xholli A, et al. Risk-reducing breast and gynecological surgery for BRCA mutation carriers: a narrative review. J Clin Med 2023;12:1422. 10.3390/jcm12041422 PMC9967164 36835955

